# More vection means more velocity storage activity: a factor in visually induced motion sickness?

**DOI:** 10.1007/s00221-018-5340-1

**Published:** 2018-08-17

**Authors:** Suzanne A. E. Nooij, Paolo Pretto, Heinrich H. Bülthoff

**Affiliations:** Department of Human Perception, Cognition and Action, Max Planck Institute for Biological Cybernetics, Max-Planck-Ring 8, 72076 Tübingen, Germany

**Keywords:** Vection, Velocity storage mechanism, Optokinetic afternystagmus, Visually induced motion sickness

## Abstract

Full-field visual rotation around the vertical axis induces a sense of self-motion (vection), optokinetic nystagmus (OKN), and, eventually, also motion sickness (MS). If the lights are then suddenly switched off, optokinetic afternystagmus (OKAN) occurs. This is due to the discharge of the velocity storage mechanism (VSM), a central integrative network that has been suggested to be involved in motion sickness. We previously showed that visually induced motion sickness (VIMS) following optokinetic stimulation is dependent on vection intensity. To shed light on this relationship, the current study investigated whether vection intensity is related to VSM activity, and thus, to the OKAN. In repetitive trials (eight per condition), 15 stationary participants were exposed to 120 s of visual yaw rotation (60°/s), followed by 90 s in darkness. The visual stimulus either induced strong vection (i.e., scene rotating normally) or weak vection (central and peripheral part moving in opposite directions). Eye movements and subjective vection intensity were continuously measured. Results showed that OKAN occurred less frequently and with lower initial magnitude in the weak-vection condition compared to the strong-vection condition. OKAN decay time constants were not significantly different. The results suggest that the stimuli that produced strong vection also enhanced the charging of the VSM. As VSM activity presumably is a factor in motion sickness, the enhanced VSM activity in our strong-vection condition hints at an involvement of the VSM in VIMS, and could explain why visual stimuli producing a strong sense of vection also elicit high levels of VIMS.

## Introduction

Motion in the whole visual field induces both optokinetic nystagmus (OKN) and a compelling sense of self-motion (vection) in stationary observers. The OKN is a reflexive eye movement driven by motion in the whole—or part of—the visual field. It helps to stabilize the visual environment on the retina and consists of a slow phase where the eye moves in the same direction as the visual stimulus, and a resetting fast phase in the opposite direction. When the lights are suddenly switched off optokinetic afternystagmus (OKAN) occurs, during which the slow-phase eye velocity (SPEV) suddenly drops and then gradually decays over time. Sometimes, a secondary afternystagmus (OKAN-II) is observed where the direction of the slow phase is reversed (Brandt et al. 1974; Cohen et al. 1977, 1981). This reversal phase is assumed to be caused by central nervous adaptation to the optokinetic stimulus (Koenig and Dichgans 1981; Furman et al. 1989). Also perceptual aftereffects occur, where the sensation of rotation continues in darkness, and may also change direction (Brandt et al. 1974).

Prolonged exposure to full-field visual motion, while being stationary, eventually evokes symptoms of visually induced motion sickness (VIMS, e.g., Crampton and Young 1953; Stern et al. 1987; Hu et al. 1989; Bonato et al. 2004; Nooij et al. 2017). In a previous study, we showed that the level of VIMS during visual yaw rotation was related to vection intensity: visual stimuli that induced strong vection induced higher levels of VIMS than visual stimuli that induced weak vection (Nooij et al. 2017). Interestingly, as we will discuss later, susceptibility to motion sickness—including VIMS—has been associated with the so-called velocity storage mechanism (VSM, Raphan et al. 1977), which is also involved in the generation of reflexive eye movements like the OKAN. In the current paper, we investigate whether the VSM is a common denominator in both vection and VIMS.

The VSM is a central integrative network that plays a role in the integration of multisensory rotational stimuli (Raphan et al. 1977) and presumably involves the nucleus prepositus hypoglossi and the medial vestibular nuclei (Leigh and Zee 2006). It can be seen as a neural leaky (i.e., non-ideal) integrator that receives input from the vestibular and visual system and governs the slow dynamics and spatial properties of compensatory eye velocity of the vestibular and optokinetic ocular reflexes (Raphan et al. 1977; Robinson 1977; Cohen et al. 1977). For example, the VSM prolongs the angular vestibulo-ocular reflex (aVOR) during head rotations at low frequencies (Raphan et al. 1979; Robinson 1977), and tends to align the eye velocity vector of the aVOR, OKN and OKAN towards the Earth vertical when the head is tilted (e.g., Angelaki and Hess 1994; Dai et al. 1991; Cohen et al. 1999). The OKAN response is considered a direct manifestation of the discharge of the VSM integrator (Raphan et al. 1977; Robinson 1977; Cohen et al. 1977), where the decay rate of slow-phase eye velocity is governed by the time constant of the VSM integrator; that is, a high time constant indicates slow decay in SPEV.

Thus far, a large body of evidence suggests that the VSM is also related to the generation of motion sickness. Dai and colleagues (2003) found that motion sickness was related to the spatio-temporal characteristics of velocity storage, as characterized by the behavior of the eye velocity vector during head rotations. They observed that the severity of motion sickness during cross-coupled Coriolis stimulation (i.e., tilting the head while rotating) was correlated with the amount of tilt of the eye velocity vector away from the spatial vertical. Furthermore, individuals susceptible to motion sickness generally show high VSM time constants (Quarck et al. 1998; Bos et al. 2002; Hoffer et al. 2003; Clement and Reschke 2018), as can be illustrated by a prolonged aVOR response during head rotation in darkness. Habituation (i.e., shortening) of the VSM time constant by repeated exposure or through medication leads to a reduction in motion sickness susceptibility (Dai et al. 2003, 2011; Young et al. 2003; Cohen et al. 2008). The relationship between motion sickness susceptibility and the VSM also extends to the visual domain, as Guo et al. (2017) showed that participants who reported sickness after exposure to full-field optokinetic stimulation in yaw exhibited longer OKAN responses (i.e., higher VSM time constants) than less susceptible participants.

Guo et al. (2017) are the first to relate properties of the VSM to VIMS susceptibility. Nevertheless, the exact role of the VSM in VIMS remains to be defined. The results of Guo and colleagues are interesting with respect to our recent findings that VIMS induced by full-field optokinetic stimulation is also correlated to vection, that is, the visually induced illusion of self-motion (Nooij et al. 2017). In that study, stationary participants were exposed to different full-field optokinetic rotational motion stimuli in yaw that evoked different levels of vection. We found that stimuli inducing a high level of vection were more nauseogenic than stimuli that suppressed vection on an individual level. The observed relationships between VIMS and the VSM (Guo et al. 2017) on the one hand, and VIMS and vection (Nooij et al. 2017) on the other, suggests that also the VSM and vection are related.

Involvement of the VSM in vection has indeed been suggested by Brandt and Dichgans (1972) and Brandt et al. (1974), who showed that oculomotor and perceptual aftereffects (i.e., OKAN and vection, resp.) following full-field optokinetic stimulation are similar in terms of duration and direction, and that both show peripheral dominance. These observations led Cohen et al. (1977) to propose that circular vection is the result of monitoring the activity in the integrators associated with OKN and OKAN. The current study aims at investigating the proposed relationship between vection and the VSM by testing the hypothesis that the OKAN, being a manifestation of the VSM, is related to the occurrence of vection. More specifically, we expect that stimuli inducing weak vection are associated with less VSM activity, hence, show a weaker OKAN than stimuli inducing strong vection. Moreover, as vection is positively related to VIMS, a comparison of OKAN responses following weak- and strong-vection-inducing stimuli sheds light on the question whether the VSM can be seen as a common denominator in vection and VIMS.

## Methods

### Ethics statement

The experiment was conducted in accordance with the Declaration of Helsinki. All participants gave their written informed consent prior to participation. The experimental protocol and consent forms were approved by the Ethical Board of the Eberhard Karls University of Tübingen.

### Participants

Data were collected on a total of 16 healthy volunteers (4 males, 12 females, mean age = 26, SD = 3), who were recruited from the local participant database. All participants were susceptible to motion sickness, meaning that they had experienced symptoms of motion sickness at least once in the last 3 years (e.g., while traveling as a passenger on a bus, car or airplane, on a fairground attraction, or while playing videogames). They were free from any known vestibular or neurological disorder and had normal vision, or corrected-to-normal vision with the aid of contact lenses.

### Experimental setup

**Fig. 1:**
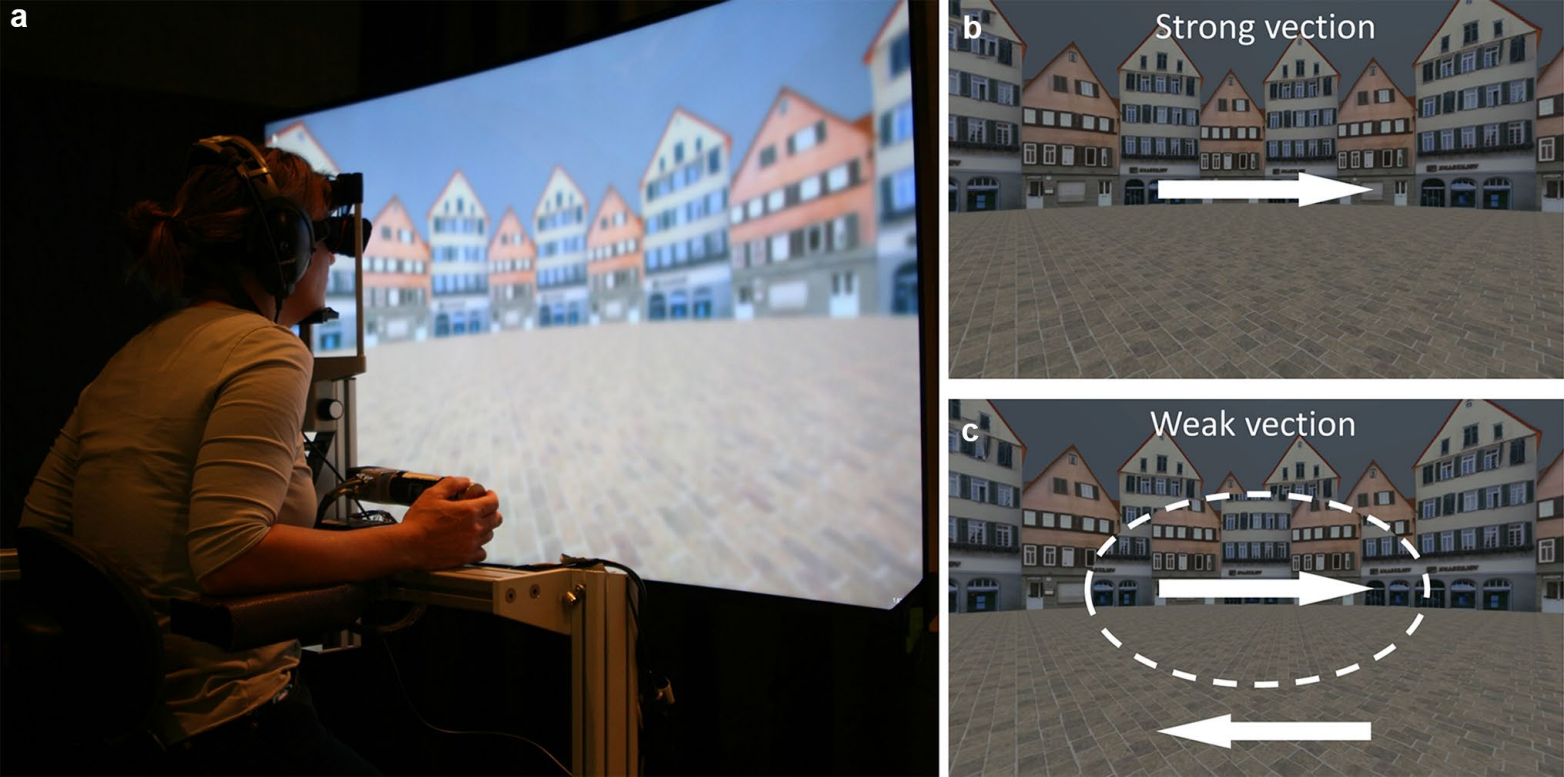
**a** Experimental setup; **b** strong-vection condition, with scene moving coherently; **c** weak-vection condition with ellipse-shaped central part (dashed line) moving opposite to the periphery. Arrows indicate direction of scene motion

The participant was seated on a height-adjustable chair in front of a large, curved TV-screen (Fig. 1a). The head was supported by a chin-and-forehead rest, whereas arm-, back- and footrests provide additional body support. The screen (Type UE65JU7500, Samsung, South Korea) was positioned at a distance of 0.47 m from the head, and provided a horizontal field of view of 120°. A light sensor attached to the right lower corner of the screen was used to detect whether the screen was on or off. Eye movements of the left eye were recorded by a head mounted, video-based eye tracking system (EyeSeeCam, Autronic Medizintechnik, Germany) at a frequency of 220 Hz. Curved black shields attached to both sides of the eye tracker goggle prevented the participant from seeing the screen edges. A rotary knob (SensoDrive GmbH, Germany) was attached to the right armrest for the continuous indication of vection strength (Nooij et al. 2017). Brown noise presented through headphones masked any environmental noise.

### Visual stimuli

As in our previous study (Nooij et al. 2017), the visual stimulus consisted of a 360° panoramic image of a circular square lined with houses. Such a naturalistic scene has been shown to induce a compelling sense of vection (Riecke et al. 2006). The scene was rotated around the vertical axis at 60°/s.

In the first experimental condition, the scene moved coherently in one direction (to the right, Fig. 1b), thus eliciting a strong sense of vection. Hence, this is termed the strong-vection condition. In the second visual condition, termed the weak-vection condition, the central and peripheral part moved in opposite directions (Fig. 1c, central part always to the right). This has been shown to reduce or even completely cancel the illusory feeling of self-motion (Nooij et al. 2017). The central part was ellipse shaped, and its size was individually fine-tuned to maximize the vection-reduction effect (see “Procedures and design”).

### Procedures and design

Prior to any data collection, the participant was informed about the general study goals and procedures and written informed consent was obtained. Subsequently, the Motion Sickness Susceptibility Questionnaire Short (MSSQ, Golding 2006) was filled out to assess the participant’s general motion sickness susceptibility. Then the participant was seated in the experimental setup and familiarized with the visual stimulus and the indication of vection with the rotary knob in two 60 s training trials. The knob could rotate over a range of 90°, and was used to obtain a continuous vection measure between the two endpoints of “No vection” (i.e., “I am stationary, the visual scene is rotating”) and “full vection” (i.e., “I am rotating and scene appears stationary”). Once familiar with the concept of vection, the weak-vection stimulus was tuned individually by determining the size of the central ellipse-shaped part that effectively suppressed vection. In an interactive procedure, the participant was first shown the effect of a small ellipse (hor. diameter = 40°, perceived self-rotation opposite to the peripheral stimulus) and a very large ellipse (hor. diameter = 110°, perceived self-rotation in the direction opposite to the central stimulus). Subsequently, the participant was asked to make size adjustments until any sensation of self-motion was minimal or absent. When satisfied, a 60-s test run was performed to verify whether the vection suppression remained stable over time. If this was not the case, additional tuning was performed. The tuning procedure generally took 5–10 min. On average, the horizontal diameter of the tuned ellipse extended over 91° (SD = 15).

After this tuning phase the first block of experimental trials was run. Each trial started with a calibration of the eye tracker, followed by 120 s of scene rotation, during which both the vection indication and eye movements were recorded. The participant was instructed to look at the visual scene, avoiding to actively track individual objects. Furthermore, the participant was instructed to indicate any occurrence of vection using the rotary knob, also in the weak-vection condition. After 120 s the screen was switched off, leaving the participant in total darkness, and recording continued for 90 s. After the trial, room lights were switched on again for another 2 min.

Both experimental conditions were repeated eight times, resulting in a total of 16 trials. Trials were grouped per condition in four blocks of four trials, with the condition alternating after each block. Condition order was counterbalanced between participants. Breaks between blocks were provided to minimize fatigue, and to keep motion sickness levels to a minimum. Motion sickness may influence alertness, which is known to affect the OKAN (Magnusson et al. 1985). To monitor motion sickness during the experiment, the participant indicated their motion sickness level before and after each block of trials. For this the numerical Fast Motion Sickness Scale was used (Keshavarz and Hecht 2011), ranging from 0 (“No symptoms”) to 20 (“I need to vomit”).

### Data analysis

Vection responses were expressed as a fraction between 0 (no vection, knob not rotated) and 1 (full vection, knob rotated 90°). As the responses were generally stable over the course of the trial, the median value during the last 90 s of stimulus presentation was taken as a measure for vection strength.

Horizontal eye velocity and acceleration were calculated from the recorded horizontal eye position using a numerical three-point differentiation and a Gaussian low-pass filter with a corner frequency of 30 Hz. OKN fast phases were removed using the algorithm of Behrens and Weiss (Behrens and Weiss 1992), and subsequently the eye velocity was filtered using a median filter with a window size of 0.5 s to obtain the slow-phase eye velocity (SPEV).

### Characterization of OKN and OKAN

OKN gain was defined as the median SPEV during the 120-s stimulus presentation, divided by the stimulus velocity (60°/s). To identify OKAN, all ocular responses were visually checked for the presence of OKAN-I, following the criteria adopted by Guo et al. (Guo et al. 2017). That is, OKAN-I was present if: (1) at least two consecutive cycles of the eye movements in complete darkness were of the OKN type with a reduced amplitude compared to that of the previous two cycles of OKN eye movements; or (2) at least four cycles of the eye movements in complete darkness were of the OKN type with at most one cycle of non-OKN-type eye movements in between. OKAN-II was present when the SPEV reached a value larger than 2°/s in the opposite direction as the OKAN-I (Brantberg 1992).

The initial amplitude of OKAN-I was defined as the median SPEV between *t* = 1.5 s and *t* = 2.5 s after ‘lights off’ (Tijssen et al. 1989). This disregards the rapid drop in SPEV occurring in the first second of OKAN. The rate of decay, reflecting the time constant of the VSM (${\tau _1}$), was estimated by fitting a single exponential function to the OKAN SPEV in the first minute of OKAN, excluding the first 2 s:

1 $$f\left( t \right)=a+b \cdot {e^{ - t/\tau _{1}}},$$

where $a$ reflects the offset and $b$ the initial amplitude of the OKAN response.

In the responses of a few participants, OKAN-II was observed, which is not accounted for by Eq. 1. The secondary nystagmus indicates the presence of a central adaptive process (Koenig and Dichgans 1981; Furman et al. 1989), and it has been shown previously for vestibular nystagmus (i.e., the aVOR) that errors in time constant estimates occur when such underlying processes are not accounted for (Demer and Robinson 1983; Ramat and Bertolini 2009). To avoid such errors, the contribution of the secondary nystagmus was estimated following the procedure used by Laurens et al. (2011) and removed before fitting Eq. 1. In short, OKAN-II was modeled as monotonously increasing exponential of the form

2 $$g\left( t \right)=m \cdot (1 - {{\text{e}}^{ - t/\tau _{2}}}).$$

The rate of increase is governed by the time constant ${\tau _2}$ and the final, maximum value is determined by the parameter $~m$. The value of ${\tau _2}$ was fixed at 30 s, matching the observation that secondary nystagmus generally peaks between 60 and 90 s. The value for $m$ was determined by the maximum OKAN-II SPEV between *t* = 50 and *t* = 70 s after ‘lights off’. Equation 2 thus allows us to model the part of OKAN-II up to the moment the maximum OKAN-II SPEV is reached, without adding additional free parameters to the fitting procedure. Model simulations showed that this procedure resulted in more accurate estimates of the VSM time constant (${\tau _1}$). Varying the value of ${\tau _2}$ had a relatively small effect on the estimate of ${\tau _1}$. Errors in the ${\tau _1}$ estimate were smaller than the consistent overestimation found when ignoring the OKAN-II. Fitting was performed using the *lsqcurvefit* function in Matlab^®^, with the upper boundary of ${\tau _1}$ set to 200 s. Trials where this boundary was reached were excluded from further analysis.

### Statistical analysis

Assumptions for data normality and homogeneity of variance were tested using Kolmogorov–Smirnov and Levene’s tests. As both assumptions were violated for the majority of the dependent variables, we used non-parametric Wilcoxon signed-rank tests to test for differences between the two visual conditions. For each participant and condition, the median value of all repetitions was used in the statistical analysis. Trial-to-trial variability was assessed by calculating the interquartile range (IQR) over all repetitions per participant and condition.

## Results

**Fig. 2:**
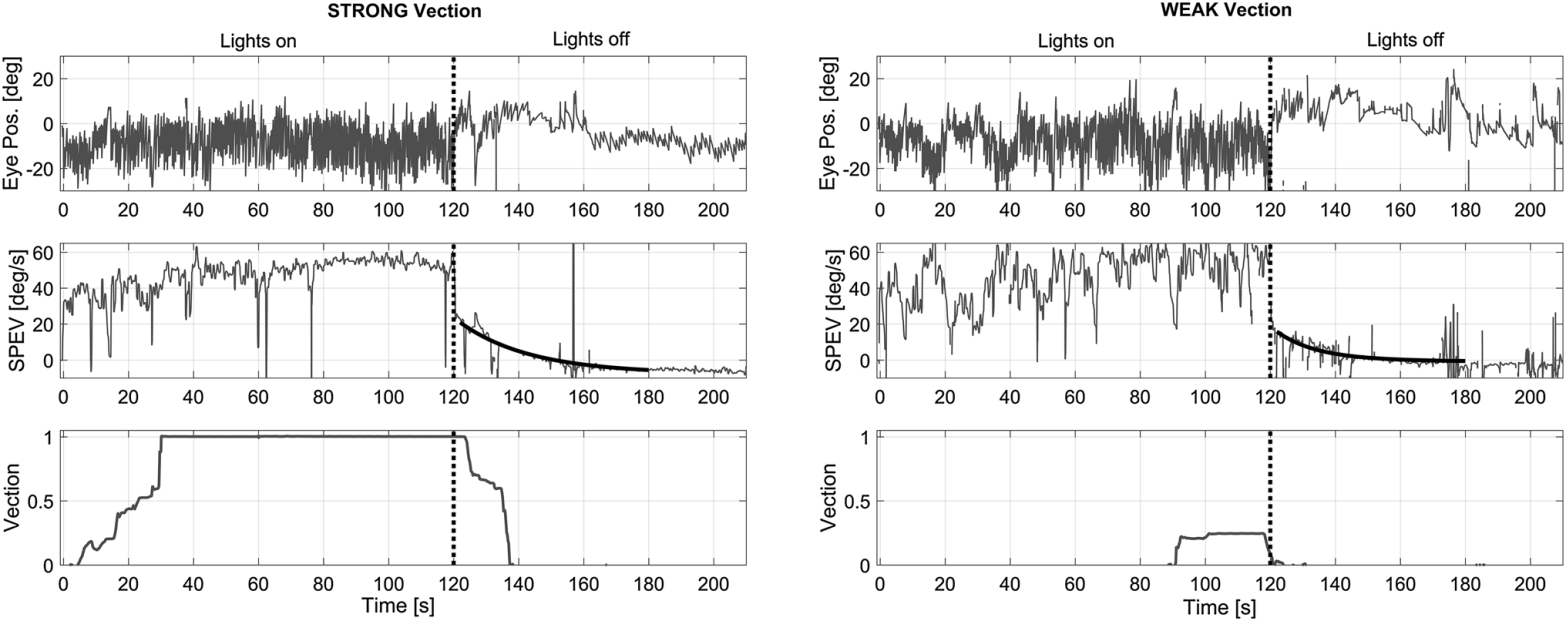
Example traces of horizontal eye position (top), slow-phase eye velocity (SPEV, center) and vection (bottom) during and after optokinetic stimulation, for the strong- (left column) and weak-vection condition (right column). The vertical dotted line indicates the moment of lights off. The exponential fit on the SPEV after lights off is overlaid

Data of 3 of the 16 participants were discarded from further analysis. One participant had to abort the experiment after the first block of trials due to motion sickness, whereas in two participants the eye movement recordings were of insufficient quality (frequent eye closure). Vection and eye movement responses of 13 participants were thus included in the statistical analysis. An example of the ocular and perceptual response for both visual conditions is shown in Fig. 2. The bottom row depicts the vection ratings, which are clearly different between conditions. After ‘lights off’ there is a sudden drop in SPEV (middle row), followed by a clear OKAN. Also a secondary OKAN is visible, where the slow phase switches direction (around *t* = 155 s). In the strong-vection condition, the perceived rotation also continues for a few seconds in darkness, whereas no after perceptual aftereffect is visible in the weak-vection condition.

### Vection strength

**Fig. 3:**
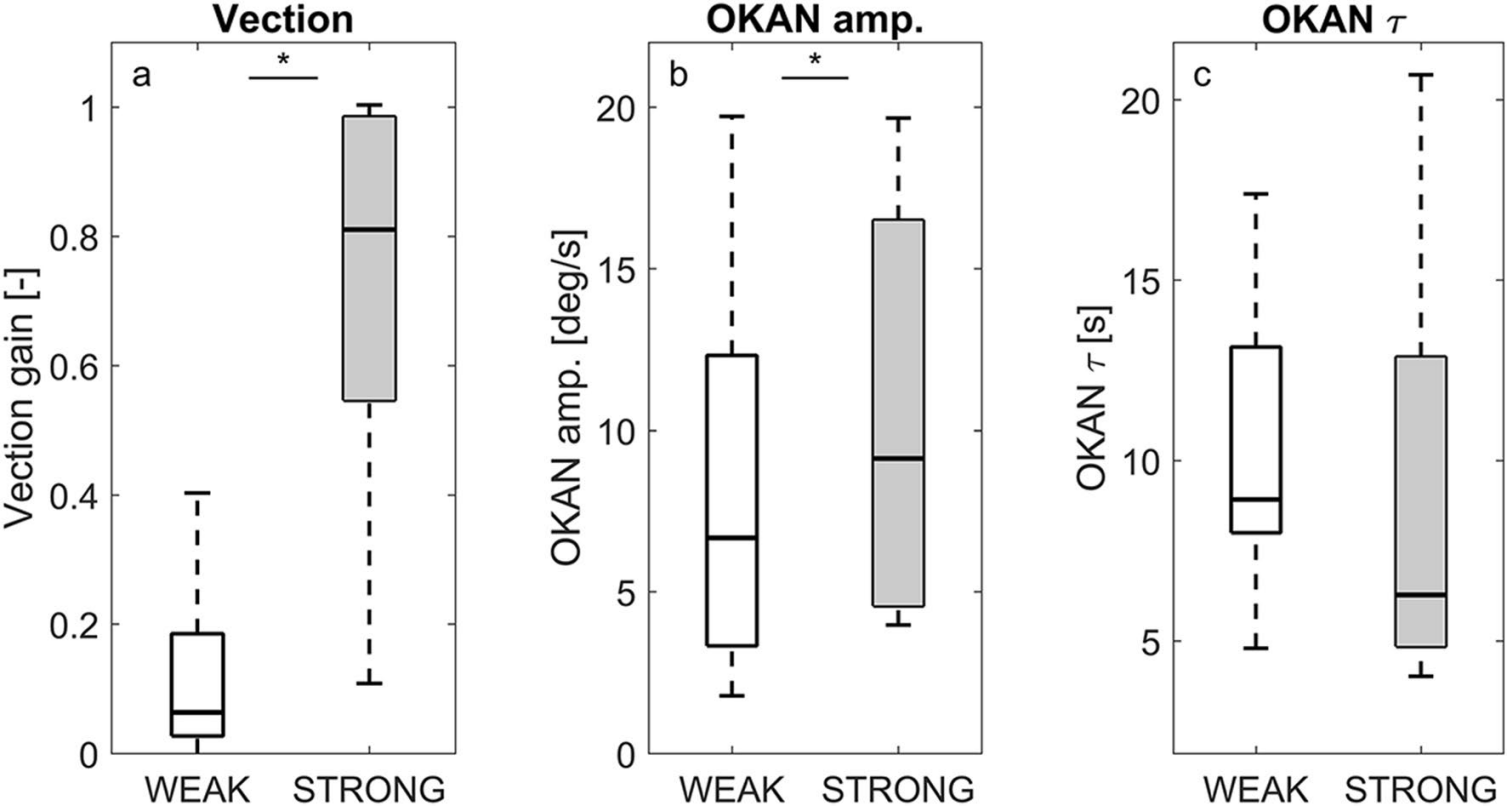
Boxplots of group results for vection strength (**a**), OKAN initial amplitude (**b**) and OKAN time constant (**c**) in the strong- and weak-vection conditions. Significant differences are indicated by an asterisk (*)

By comparing the vection strength of the two experimental conditions, we verified that the visual stimulus was successful in manipulating vection (Fig. 3a). As anticipated, vection ratings were high in strong-vection condition (Med = 0.8, IQR = 0.43) and low in the weak-vection condition (Med = 0.1, IQR = 0.2). This difference was significant (*T* = 91, *p* < 0.001). The vection ratings were fairly consistent over repeated trials. Over all participants, the median IQR over repeated trials was 0.12 in both conditions. Vection ratings were also stable *within* one trial, with a median IQR of 0.057.

As is also visible in Fig. 2, in most cases the sensation of rotation continued for several seconds after the lights were switched off. Median duration of this vection aftereffect (VAE) was 12.8 s (IQR = 15.9) for the strong-vection condition, and 3.8 s (IQR = 8.9) in the weak-vection condition (*T* = 89, *p* = 0.001).

### Ocular responses

Visual inspection of the data revealed that the OKAN response was observed in 82% of all trials in the strong-vection condition, vs. 62% of the trials in the weak-vection condition. A secondary OKAN response was observed in 4 of the 13 participants, accounting for 24% of the trials (comparable occurrence in both conditions).

For the trials where OKAN was present, the response was characterized by its initial amplitude and a decay time constant (see “Methods”). Figure 3b, c shows a summary of these data, and it can be seen that the initial amplitude was significantly lower in the weak-vection condition (*T* = 59, *p* = 0.019). In the strong-vection condition, the median initial amplitude was 9.1°/s (IQR = 12.2), whereas it was 6.7°/s (IQR = 9.0) in the weak-vection condition. Trial-to-trial variability in the initial amplitude, as expressed by the IQR over the eight repetitions, was 2.5°/s for both conditions (median value over all participants).

**Fig. 4:**
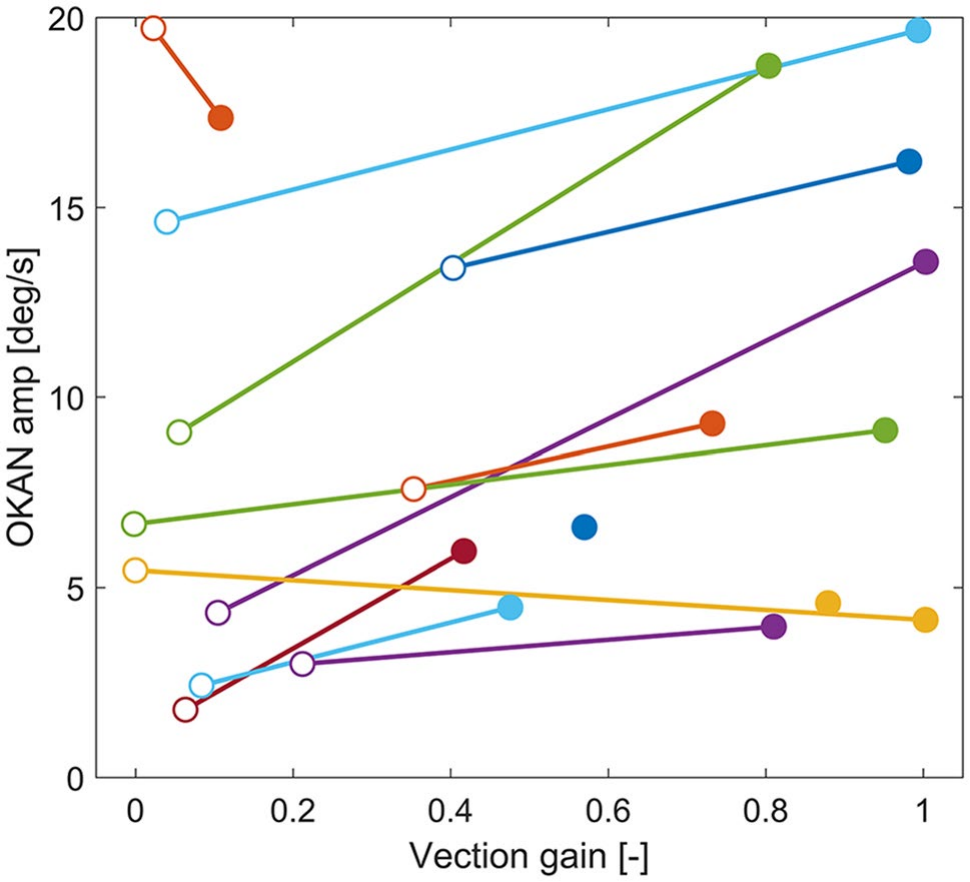
Individual vection gains vs. OKAN initial amplitude for the weak- (open symbols) and strong (filled symbols)-vection condition

In Fig. 4, the individual data for the initial OKAN amplitude are plotted against the vection gain. For the majority of participants, a decreased vection gain was indeed accompanied by a decreased OKAN amplitude. On an individual level, we observed a moderate but non-significant correlation between the individual decrease in OKAN amplitude and the decrease in vection gain (*r* = 0.39, *p* = 0.11). Vection did not prove to be a prerequisite for the OKAN to occur, as some participants experienced a complete suppression of vection in the weak-vection condition, but still showed a clear OKAN response. Also the opposite occurred, that is, no OKAN response in the presence of vection.

In the comparisons presented above, the initial OKAN amplitude is based on the trials where the OKAN was identified by visual inspection. As mentioned above, the OKAN occurred less frequently in the weak-vection condition. The difference in SPEV 2 s after ‘lights off’ increases when all trials are included in the comparison: 8.9°/s in the strong-vection condition vs. 4.3°/s in the weak-vection condition, respectively (*T* = 83, *p* = 0.006).

In contrast to the initial amplitude, the decay time constant of OKAN was not affected by the experimental condition (*T* = 22, *p* = 0.6). The median time constant equaled 6.2 s (IQR = 9.7) in the strong-vection condition and 8.9 s (IQR = 5.2) in the weak-vection condition, with considerable variability between trials. The median IQR over all repetitions equal to 5.8 and 8.8 s for the strong- and weak-vection condition, respectively.

The OKN response (i.e., *during* optokinetic stimulation) was also affected by the visual condition. Although the group median was comparable (strong vection: Med = 0.81, IQR = 0.36; weak vection: Med = 0.80, IQR = 0.50), the majority of participants showed a lower gain in the weak-vection condition. The difference was small but significant (Med = − 0.06, IQR = 0.11; *T* = 75, *p* = 0.040). Trial-to-trial variability in the OKN gain was comparable to that of the vection ratings, the median IQR over all repetitions equal to 0.11 (strong-vection) and 0.14 (weak-vection).

### Motion sickness

The repetitive short trials to measure OKAN were not intended to induce VIMS, and in general, motion sickness scores were low. The median FMS score equaled 2 on the 20-point scale, but scores ranged from 0 to 9. To determine any effect of the visual condition on motion sickness, we calculated the mean difference score between start and end of each block per participant. This difference score was significantly smaller for the weak-vection condition (*T* = 32.5, *p* = 0.047), indicating that this condition was less provocative than the strong-vection condition.

### Correlations between OKAN, motion sickness and vection

**Fig. 5:**
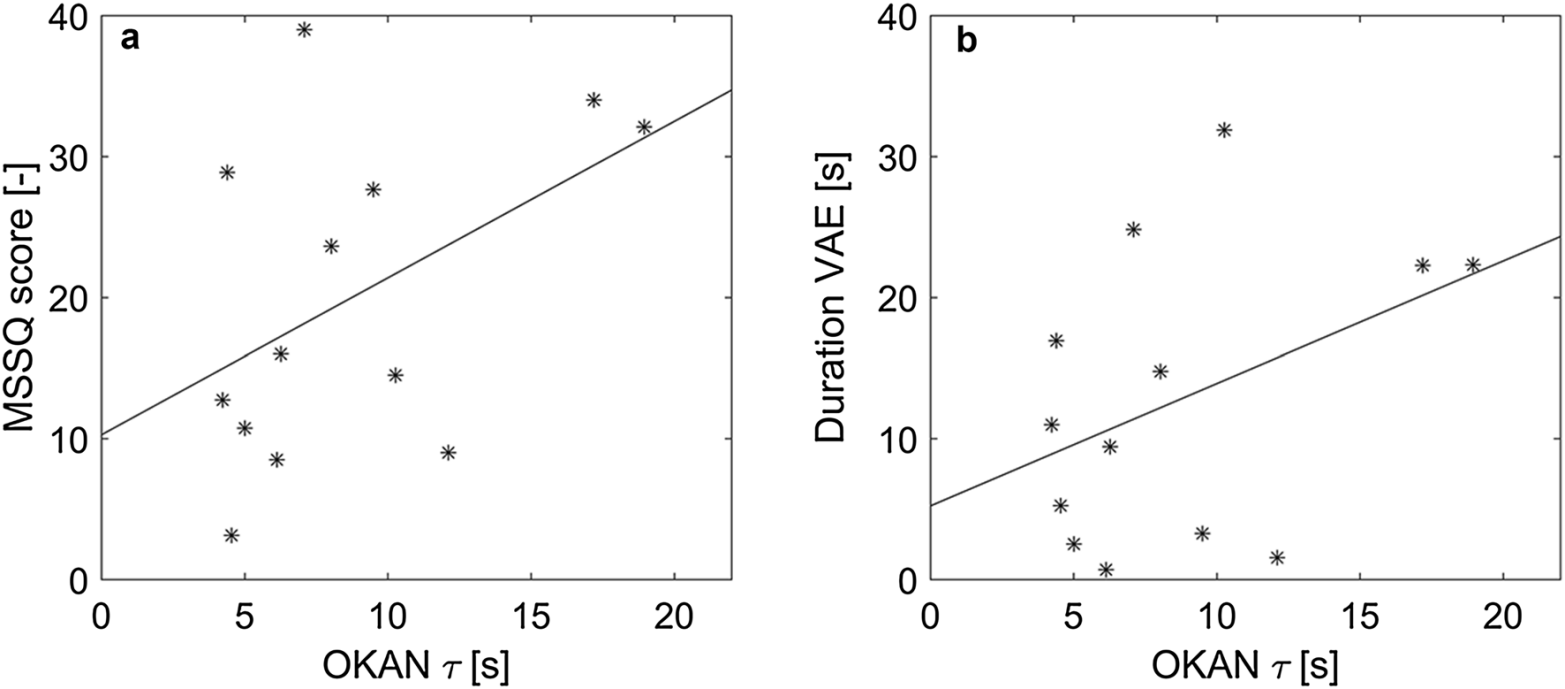
Relationship between OKAN time constant and MSSQ (**a**) and the duration of the vection aftereffect (**b**)

Previous studies indicated a positive relationship between the velocity storage time constant and motion sickness susceptibility (Quarck et al. 1998; Bos et al. 2002; Hoffer et al. 2003; Clement and Reschke 2018; Guo et al. 2017). As our focus lied on measurement of OKAN and not on inducing motion sickness, we used the MSSQ scores (and not the FMS scores) as a measure for individual motion sickness susceptibility, and compared those with the individual OKAN time constants (Fig. 5a). There was indeed a moderate positive correlation between the two variables, of which the significance was just above the 0.05 level (*r* = 0.46, *p* = 0.054). No significant correlation was found between the OKAN initial amplitude and the MSSQ (*r* = 0.22, *p* = 0.23).

There was also moderate correlation between the OKAN time constant and the duration of the vection aftereffect (VAE) in the strong-vection condition, albeit not significant (*r* = 0.41, *p* = 0.08, Fig. 5b). A relationship between these variables would be in line with Brandt and Dichgans (1972) and Brandt et al. (1974), who reported similar time courses of the perceptual and oculomotor aftereffects. Note, however, that in our results time constant and VAE duration are in the similar order of magnitude. That means that the OKAN generally outlasted the perceptual response, requiring about three times the time constant to decay to zero.

## Discussion

Optokinetic stimulation over the whole field of view induces eye movements, a compelling sense of vection, and eventually may evoke motion sickness. In this paper, we investigated whether the optokinetic afternystagmus (OKAN), occurring *after* a period of optokinetic stimulation, was related to the occurrence of vection *during* the optokinetic stimulation. The OKAN response is a manifestation of the velocity storage mechanism (VSM) and both the VSM and vection have been associated to (visually induced) motion sickness. Since stimuli that produce strong vection are also known to be nauseogenic, we hypothesized that these stimuli would also show a higher level of VSM activity, hence a stronger OKAN response, than stimuli that induce lower levels of vection.

Vection intensity was successfully manipulated by having the visual stimulus rotating congruently in the same direction (strong vection) or by having the central and peripheral part rotating in opposite directions (weak vection). The OKAN response was characterized by its initial amplitude and a decay time constant, and values observed in our study were in agreement with those of others (Cohen et al. 1981; Guo et al. 2017; Jell et al. 1984; Tijssen et al. 1989; Fletcher et al. 1990; Clement and Lathan 1991; Laurens et al. 2011). As it is well known that the OKAN response is subject to a large trial-to-trial variability, e.g., by changing levels of alertness (Magnusson et al. 1985), the results were based on data of eight repetitions per condition. Data of Tijssen et al. (1989) showed that this number of repetitions is sufficient to get a reliable estimate for both the initial amplitude and time constant.

The results confirm our hypothesis that the OKAN response is dependent on the visual stimulus that affected vection intensity. In the weak-vection condition, OKAN occurred less frequently and with a lower initial amplitude than in the strong-vection condition. The time constant was not affected by the visual stimulus. These results suggest a decrease in VSM activity in the weak-vection condition. The model of Cohen et al. (1977) and Raphan et al. (1977) describes the overall ocular response to visual and vestibular stimulation in terms of a direct pathway, responsible for the fast dynamics, and an indirect pathway, responsible for the slow dynamics of the response. The latter contains the VSM integrator. The initial amplitude of the OKAN is determined by both the VSM time constant, the overall pathway gain, and, of course, the visual input to the VSM (see Cohen et al. 1977 for a mathematical derivation). We did not find any evidence for a change in time constant between the conditions, which is in line with the notion that the time constant can be regarded as a characteristic of the system, rather than being dependent on the visual input. A reduction in OKAN amplitude could, thus, hint at a decrease in the overall gain of the VSM pathway, resulting in lower VSM activity in our weak-vection condition. As the VSM also contributes to the OKN gain *during* optokinetic stimulation, a lower VSM pathway gain is also consistent with the lower OKN gain observed in the weak-vection condition. Alternatively, a lower initial OKAN amplitude could be explained by a lower input to the VSM. This would indicate that the differences in movement direction between the central and peripheral part in the weak-vection condition are already taken into account before entering the velocity storage pathway. Both explanations are in agreement with a decrease in overall VSM activity.

The results are partly in line with the notion that the OKAN and vection both reflect activity in the VSM integrator during visual rotation (Cohen et al. 1977). A lower vection intensity during the optokinetic stimulation was associated with lower OKAN amplitude, and, on an individual level, there was a moderate (but non-significant) positive correlation between the change in vection and the change in OKAN amplitude. Furthermore, there was a moderate (but non-significant) correlation between the duration of the perceptual aftereffect and the OKAN time constant. On the other hand, participants who experienced a complete cancellation of vection still showed (reduced) OKAN responses, and, vice versa, the presence of vection was no guarantee for the presence of an OKAN response. This suggests that, if the VSM indeed affects both responses, it has a different contribution to perception and eye movements. This was also proposed by Bertolini et al. (2011), who showed that perceptual and oculomotor responses during head rotations in darkness could be modelled by the same VSM pathway, but assuming different relative weights. In addition, vection has been found to be affected by cognitive factors (Riecke et al. 2006; Golding and Doolan 2012), whereas the OKAN is known to be altered by attention (Magnusson et al. 1985). Such influences might further increase the dissociation between perceptual and oculomotor responses.

The findings of this study may shed a different light on the causal mechanism of motion sickness resulting from prolonged visual rotation (VIMS), e.g., when using an optokinetic drum (e.g., Crampton and Young 1953; Stern et al. 1987; Hu et al. 1989; Bonato et al. 2004; Nooij et al. 2017). Our previous study linked the occurrence of VIMS to vection. Participants who experienced a higher vection intensity were not necessarily also more susceptible to VIMS, but we found that stimuli inducing strong vection were more nauseogenic (Nooij et al. 2017). Traditionally, the occurrence of VIMS during vection is explained in terms of sensory conflict, as the visually induced sense of rotation is not corroborated by congruent vestibular information (Reason and Brandt 1975). We argued that this does not sufficiently explain VIMS, as this conflict is only assumed to be present at the start of the stimulus, when vection is still building up (Nooij et al. 2017). As the vestibular system is not responsive to constant velocity rotation, the conflict is expected to disappear when full vection is reached. This usually occurs within the first minute, whereas VIMS symptoms take much longer to build up. The results of the current study hint at another mechanism that might be involved here: the VSM. As stated in the introduction, involvement of the VSM in motion sickness is suggested by the positive correlation between motion sickness susceptibility and the VSM time constant (Quarck et al. 1998; Bos et al. 2002; Hoffer et al. 2003; Clement and Reschke 2018) and the finding that shortening of the time constant by habituation or medication is accompanied by a reduction in susceptibility (Dai et al. 2003, 2011; Young et al. 2003; Cohen et al. 2008). Guo et al. found that this relationship was not restricted to motion sickness due to physical motion, but also holds for VIMS (Guo et al. 2017). This was confirmed in the current study, although the observed correlation was only moderate. This is likely due to the fact that we measured motion sickness susceptibility using a questionnaire, which provides less-specific responses than a direct measurement. Besides confirming the relationship between motion sickness susceptibility and the VSM time constant, the main contribution of this paper is that it relates VSM activity to properties of the *visual stimulus*: The weak-vection stimulus reduced the amount to which the integrator was charged. Combined with the earlier finding that vection intensity affects VIMS (Nooij et al. 2017), these results provide further evidence for a link between the VSM and VIMS.

If the OKAN amplitude relates to VIMS, one might expect that participants with a high OKAN amplitude also show a higher motion sickness susceptibility. The results did not demonstrate such a correlation, which suggests that VSM activity per se is not indicative for motion sickness susceptibility. This is in line with other studies showing that the gain of the aVOR, which is also affected by VSM activity, is not correlated with motion sickness susceptibility (Quarck et al. 1998; Clement and Reschke 2018). Instead, our data suggest that the relationship between OKAN amplitude and VIMS is likely on the level of the stimulus: the stimulus that induced less VSM activity is also known to be less provocative than a stimulus inducing stronger VSM activity. The same pattern was found in our pervious study where a link between VIMS and vection was demonstrated (Nooij et al. 2017). Therein, the participants with the highest vection ratings were not necessarily the most sick, but when compared within participants, stimuli that induced stronger vection were also more provocative in terms of VIMS.

In conclusion, the results of this study suggest a role for the VSM in VIMS. There are, however, still many open questions concerning the exact underlying mechanism. Dai and colleagues proposed that motion sickness was mediated through the orientation properties of the VSM that tend to align the eye velocity vector towards the spatial vertical (Dai et al. 2003). Alternatively, it has been proposed that the VSM plays a more general, functional role in the estimation of the vertical (Green and Angelaki 2003; Laurens and Angelaki 2011), and perception of the spatial vertical indeed is considered to be an important factor in motion sickness (Bles et al. 1998). The latter is illustrated by the observation that rotating the head and body around an off-vertical axis rotations is very provocative, whereas vertical axis rotations are not (e.g., Leger et al. 1981). Although these examples illustrate the importance of the spatial vertical in motion sickness, such explanations do not account for situations where the vertical is not at stake, like the visual vertical axis rotation applied in the current study. Regardless of the fact that this is a relatively benign stimulus, its provocative nature has been demonstrated many times. Our findings hint at a role for the VSM therein, and may help guide further research to clarify the exact underlying mechanism.
